# 2-Chloro-*N*-(2,3-dichloro­phen­yl)benzamide

**DOI:** 10.1107/S1600536808018679

**Published:** 2008-06-25

**Authors:** B. Thimme Gowda, Sabine Foro, B. P. Sowmya, Hartmut Fuess

**Affiliations:** a Department of Chemistry, Mangalore University, Mangalagangotri 574 199, Mangalore, India; b Institute of Materials Science, Darmstadt University of Technology, Petersenstrasse 23, D-64287 Darmstadt, Germany

## Abstract

Two independent mol­ecules comprise the asymmetric unit in the title compound, C_13_H_8_Cl_3_NO, each with the amide N—H and C=O bonds *trans* to each other. The mol­ecules are linked into chains through inter­molecular N—H⋯O and N—H⋯Cl hydrogen bonds.

## Related literature

For related literature, see: Gowda *et al.* (2003 ▶, 2007 ▶, 2008 ▶).
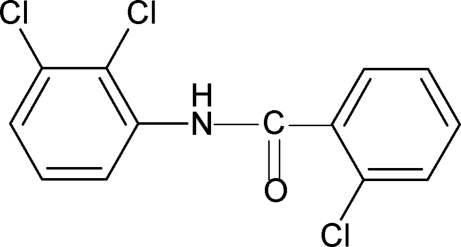


## Experimental

### 

#### Crystal data

C_13_H_8_Cl_3_NO
*M* *_r_* = 300.55
Monoclinic, 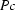

*a* = 12.310 (1) Å
*b* = 7.8307 (6) Å
*c* = 14.407 (2) Å
β = 111.52 (1)°
*V* = 1292.0 (2) Å^3^
*Z* = 4
Mo *K*α radiation
μ = 0.69 mm^−1^
*T* = 299 (2) K
0.75 × 0.75 × 0.18 mm

#### Data collection

Oxford Diffraction Xcalibur diffractometer with a Sapphire CCD detector
Absorption correction: multi-scan (*CrysAlis RED*; Oxford Diffraction, 2007 ▶) *T*_min_ = 0.624, *T*_max_ = 0.885
5882 measured reflections
3936 independent reflections
3430 reflections with *I* > 2σ(*I*)
*R*_int_ = 0.014

#### Refinement

*R*[*F* ^2^ > 2σ(*F* ^2^)] = 0.032
*wR*(*F* ^2^) = 0.104
*S* = 1.14
3936 reflections
326 parameters
2 restraints
H-atom parameters constrained
Δρ_max_ = 0.29 e Å^−3^
Δρ_min_ = −0.28 e Å^−3^
Absolute structure: (Flack, 1983 ▶), 365 Friedel pairs
Flack parameter: 0.12 (5)

### 

Data collection: *CrysAlis CCD* (Oxford Diffraction, 2007 ▶); cell refinement: *CrysAlis RED* (Oxford Diffraction, 2007 ▶); data reduction: *CrysAlis RED*; program(s) used to solve structure: *SHELXS97* (Sheldrick, 2008 ▶); program(s) used to refine structure: *SHELXL97* (Sheldrick, 2008 ▶); molecular graphics: *PLATON* (Spek, 2003 ▶); software used to prepare material for publication: *SHELXL97* .

## Supplementary Material

Crystal structure: contains datablocks I, global. DOI: 10.1107/S1600536808018679/tk2277sup1.cif

Structure factors: contains datablocks I. DOI: 10.1107/S1600536808018679/tk2277Isup2.hkl

Additional supplementary materials:  crystallographic information; 3D view; checkCIF report

## Figures and Tables

**Table 1:** Hydrogen-bond geometry (Å, °)

*D*—H⋯*A*	*D*—H	H⋯*A*	*D*⋯*A*	*D*—H⋯*A*
N1—H1*N*⋯O2	0.86	2.16	2.850 (3)	137
N1—H1*N*⋯Cl3	0.86	2.64	3.114 (3)	116
N2—H2*N*⋯O1^i^	0.86	2.05	2.896 (3)	167

Symmetry code: (i) 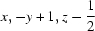.

**Fig. 1.**
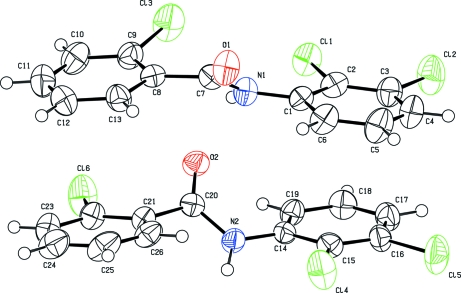
Molecular structure of (I) showing the atom labeling scheme. The displacement ellipsoids are drawn at the 50% probability level.

**Fig. 2.**
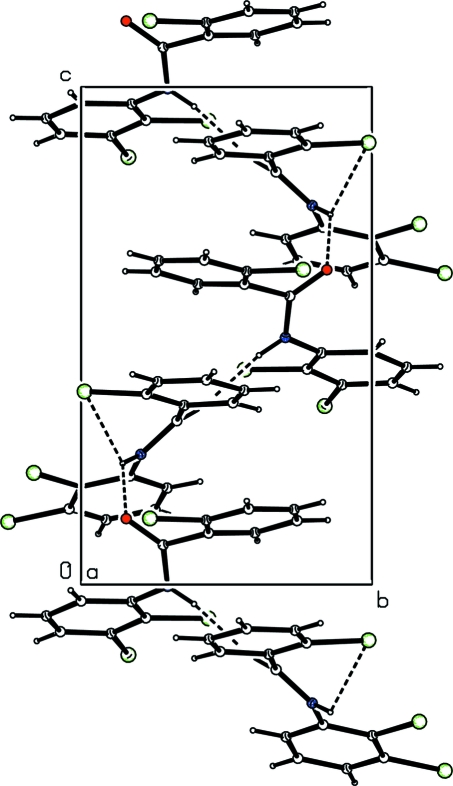
Molecular packing of (I) with hydrogen bonding shown as dashed lines.

## supplementary crystallographic information

### Comment

In the present work, the structure of 2-chloro-*N*-(2,3-dichlorophenyl)- benzamide (I, N23DCP2CBA) has been determined to explore the effect of substituents on the structures of benzanilides (Gowda *et al.*, 2003; 2007; 2008). The amide N—H and C=O bonds in each of the two molecules comprising the crystallographic asymmetric unit are *trans* to each other (Fig. 1), similar to that observed in 2-chloro-*N*-(phenyl)-benzamide (NP2CBA) (Gowda *et al.*, 2003), 2-chloro-*N*-(2-chlorophenyl)-benzamide (N2CP2CBA) (Gowda *et al.*, 2007), and 2-chloro-*N*-(3-chlorophenyl)-benzamide (N3CP2CBA) (Gowda *et al.*, 2008). In one of the molecules, the conformation of the N—H bond is *syn* to both the *ortho* and *meta*-chloro groups in the aniline ring and of the C=O bond group is also *syn* to the *ortho*-chloro group in the benzoyl ring. By contrast, the conformations of these bonds in the second independent molecule are intermediate between *syn* and *anti* to the respective groups. The above conformations are in contrast to the *syn* conformations of both the amide N—H and C=O bonds, with respect to the *ortho*-chloro groups in the benzoyl and aniline rings, respectively, observed in N2CP2CBA (Gowda *et al.*, 2007). Further, in N3CP2CBA, the conformation of the C=O bond is *syn* to the *ortho*-chloro group of the benzoyl ring, while the N—H bond is *anti* to the *meta*-chloro group of the aniline ring (Gowda *et al.*, 2008). The –NHCO– group makes the dihedral angles of 42.24 (14)° (molecule 1), 48.89 (12)° (molecule 2), and 35.31 (19)° (molecule 1), 41.88 (13)° (molecule 2) with the benzoyl and aniline rings, respectively. The benzoyl and aniline rings form the dihedral angles of 12.30 (10)° (molecule 1) and 7.25 (9)°) (molecule 2). In the crystal structure of (I), the molecules are linked by intermolecular N—H···O and N—H···Cl hydrogen bonds (Table 1) forming chains running along the *a* axis, as shown in Fig. 2.

### Experimental

Compound (I) was prepared according to the literature method (Gowda *et al.*, 2003). The purity of the compound was confirmed by melting point, and IR and NMR spectra. Single crystals were obtained from an ethanolic solution of (I)

### Refinement

The H atoms were positioned with idealized geometry using a riding model with C—H = 0.93 Å and N—H = 0.86 Å, and with *U*_iso_(H) = 1.2*U*_eq_(N, C).

### Crystal data

**Table:**

C_13_H_8_Cl_3_NO	*F*_000_ = 608
*M**_r_* = 300.55	*D*_x_ = 1.545 Mg m^−^^3^
Monoclinic, *P**c*	Mo *K*α radiation λ = 0.71073 Å
Hall symbol: P -2yc	Cell parameters from 3409 reflections
*a* = 12.310 (1) Å	θ = 2.6–27.5º
*b* = 7.8307 (6) Å	µ = 0.69 mm^−^^1^
*c* = 14.407 (2) Å	*T* = 299 (2) K
β = 111.52 (1)º	Thick plate, colourless
*V* = 1292.0 (2) Å^3^	0.75 × 0.75 × 0.18 mm
*Z* = 4	

### Data collection

**Table:**

Oxford Diffraction Xcalibur diffractometer with a Sapphire CCD detector	3936 independent reflections
Radiation source: fine-focus sealed tube	3430 reflections with *I* > 2σ(*I*)
Monochromator: graphite	*R*_int_ = 0.014
*T* = 299(2) K	θ_max_ = 26.4º
Rotation method data acquisition using ω and φ scans	θ_min_ = 2.6º
Absorption correction: multi-scan(CrysAlis RED; Oxford Diffraction, 2007)	*h* = −15→13
*T*_min_ = 0.624, *T*_max_ = 0.885	*k* = −9→9
5882 measured reflections	*l* = −15→18

### Refinement

**Table:**

Refinement on *F*^2^	Hydrogen site location: inferred from neighbouring sites
Least-squares matrix: full	H-atom parameters constrained
*R*[*F*^2^ > 2σ(*F*^2^)] = 0.032	*w* = 1/[σ^2^(*F*_o_^2^) + (0.0715*P*)^2^ + 0.0104*P*] where *P* = (*F*_o_^2^ + 2*F*_c_^2^)/3
*wR*(*F*^2^) = 0.104	(Δ/σ)_max_ = 0.003
*S* = 1.14	Δρ_max_ = 0.29 e Å^−^^3^
3936 reflections	Δρ_min_ = −0.28 e Å^−^^3^
326 parameters	Extinction correction: SHELXL97 (Sheldrick, 2008), Fc^*^=kFc[1+0.001xFc^2^λ^3^/sin(2θ)]^-1/4^
2 restraints	Extinction coefficient: 0.0118 (14)
Primary atom site location: structure-invariant direct methods	Absolute structure: (Flack, 1983), 365 Friedel pairs
Secondary atom site location: difference Fourier map	Flack parameter: 0.12 (5)

### Special details

**Table:**

Geometry. All e.s.d.'s (except the e.s.d. in the dihedral angle between two l.s. planes) are estimated using the full covariance matrix. The cell e.s.d.'s are taken into account individually in the estimation of e.s.d.'s in distances, angles and torsion angles; correlations between e.s.d.'s in cell parameters are only used when they are defined by crystal symmetry. An approximate (isotropic) treatment of cell e.s.d.'s is used for estimating e.s.d.'s involving l.s. planes.
Refinement. Refinement of *F*^2^ against ALL reflections. The weighted *R*-factor *wR* and goodness of fit *S* are based on *F*^2^, conventional *R*-factors *R* are based on *F*, with *F* set to zero for negative *F*^2^. The threshold expression of *F*^2^ > σ(*F*^2^) is used only for calculating *R*-factors(gt) *etc*. and is not relevant to the choice of reflections for refinement. *R*-factors based on *F*^2^ are statistically about twice as large as those based on *F*, and *R*- factors based on ALL data will be even larger.

### Fractional atomic coordinates and isotropic or equivalent isotropic displacement parameters (Å^2^)

**Table:**

	*x*	*y*	*z*	*U*_iso_*/*U*_eq_	
Cl1	0.80355 (8)	−0.16289 (10)	0.22458 (7)	0.0542 (2)	
Cl2	0.53668 (10)	−0.25096 (14)	0.12596 (9)	0.0739 (3)	
Cl3	1.08381 (10)	0.01157 (12)	0.38808 (9)	0.0745 (3)	
O1	0.8488 (2)	0.4258 (3)	0.36026 (18)	0.0537 (6)	
N1	0.8566 (2)	0.2051 (3)	0.26179 (19)	0.0411 (6)	
H1N	0.9028	0.1426	0.2437	0.049*	
C1	0.7362 (3)	0.1682 (4)	0.2183 (2)	0.0359 (6)	
C2	0.7006 (3)	−0.0018 (4)	0.1965 (2)	0.0420 (7)	
C3	0.5819 (3)	−0.0414 (5)	0.1521 (3)	0.0493 (8)	
C4	0.4996 (3)	0.0858 (5)	0.1303 (3)	0.0566 (9)	
H4	0.4206	0.0589	0.1018	0.068*	
C5	0.5342 (4)	0.2543 (5)	0.1510 (3)	0.0597 (10)	
H5	0.4783	0.3403	0.1357	0.072*	
C6	0.6515 (3)	0.2954 (5)	0.1941 (3)	0.0498 (8)	
H6	0.6738	0.4091	0.2071	0.060*	
C7	0.9063 (3)	0.3294 (4)	0.3292 (2)	0.0388 (7)	
C8	1.0362 (3)	0.3540 (4)	0.3601 (2)	0.0379 (7)	
C9	1.1208 (3)	0.2248 (5)	0.3829 (3)	0.0478 (8)	
C10	1.2384 (4)	0.2639 (6)	0.4080 (3)	0.0613 (10)	
H10	1.2936	0.1769	0.4226	0.074*	
C11	1.2727 (3)	0.4310 (6)	0.4112 (3)	0.0629 (10)	
H11	1.3513	0.4569	0.4276	0.076*	
C12	1.1924 (4)	0.5604 (5)	0.3904 (3)	0.0576 (9)	
H12	1.2164	0.6735	0.3931	0.069*	
C13	1.0746 (3)	0.5220 (4)	0.3654 (3)	0.0455 (7)	
H13	1.0206	0.6104	0.3519	0.055*	
Cl4	0.61868 (9)	0.43793 (11)	−0.06792 (10)	0.0700 (3)	
Cl5	0.41892 (8)	0.16238 (16)	−0.14120 (9)	0.0747 (3)	
Cl6	1.22481 (9)	0.23655 (14)	0.13296 (10)	0.0727 (3)	
O2	0.9822 (2)	0.1549 (3)	0.13253 (16)	0.0436 (5)	
N2	0.8590 (2)	0.2974 (3)	−0.00345 (19)	0.0400 (6)	
H2N	0.8503	0.3882	−0.0390	0.048*	
C14	0.7697 (3)	0.1737 (4)	−0.0342 (2)	0.0351 (6)	
C15	0.6526 (3)	0.2227 (4)	−0.0670 (2)	0.0412 (7)	
C16	0.5648 (3)	0.1028 (4)	−0.0983 (2)	0.0422 (7)	
C17	0.5907 (3)	−0.0713 (5)	−0.0947 (3)	0.0485 (8)	
H17	0.5314	−0.1525	−0.1133	0.058*	
C18	0.7064 (3)	−0.1199 (4)	−0.0630 (3)	0.0471 (8)	
H18	0.7250	−0.2351	−0.0618	0.056*	
C19	0.7949 (3)	−0.0002 (4)	−0.0329 (2)	0.0413 (7)	
H19	0.8723	−0.0359	−0.0115	0.050*	
C20	0.9589 (3)	0.2819 (4)	0.0796 (2)	0.0347 (6)	
C21	1.0337 (3)	0.4382 (4)	0.1036 (2)	0.0364 (6)	
C22	1.1550 (3)	0.4300 (4)	0.1294 (2)	0.0432 (7)	
C23	1.2224 (3)	0.5764 (6)	0.1521 (3)	0.0598 (9)	
H23	1.3026	0.5697	0.1680	0.072*	
C24	1.1699 (4)	0.7327 (5)	0.1510 (3)	0.0635 (11)	
H24	1.2149	0.8316	0.1656	0.076*	
C25	1.0513 (4)	0.7428 (5)	0.1285 (3)	0.0604 (10)	
H25	1.0168	0.8479	0.1297	0.072*	
C26	0.9839 (3)	0.5976 (4)	0.1043 (2)	0.0464 (8)	
H26	0.9037	0.6060	0.0882	0.056*	

### Atomic displacement parameters (Å^2^)

**Table:**

	*U*^11^	*U*^22^	*U*^33^	*U*^12^	*U*^13^	*U*^23^
Cl1	0.0515 (5)	0.0378 (4)	0.0674 (5)	0.0092 (4)	0.0148 (4)	−0.0055 (4)
Cl2	0.0619 (7)	0.0521 (5)	0.0969 (8)	−0.0162 (5)	0.0164 (6)	−0.0149 (5)
Cl3	0.0614 (6)	0.0395 (5)	0.1037 (8)	0.0091 (5)	0.0080 (6)	0.0133 (5)
O1	0.0443 (14)	0.0479 (13)	0.0663 (14)	0.0016 (11)	0.0173 (11)	−0.0199 (12)
N1	0.0360 (14)	0.0392 (13)	0.0486 (14)	0.0038 (11)	0.0162 (11)	−0.0078 (11)
C1	0.0353 (15)	0.0343 (15)	0.0375 (15)	0.0061 (12)	0.0127 (12)	−0.0007 (12)
C2	0.0448 (17)	0.0418 (17)	0.0389 (16)	0.0093 (15)	0.0149 (13)	−0.0026 (13)
C3	0.0467 (19)	0.0471 (18)	0.0511 (19)	−0.0055 (16)	0.0147 (15)	−0.0106 (15)
C4	0.0379 (19)	0.060 (2)	0.064 (2)	−0.0022 (17)	0.0095 (15)	−0.0069 (18)
C5	0.043 (2)	0.053 (2)	0.074 (3)	0.0176 (17)	0.0104 (17)	−0.0041 (18)
C6	0.050 (2)	0.0376 (17)	0.055 (2)	0.0071 (15)	0.0115 (15)	−0.0063 (15)
C7	0.0414 (17)	0.0326 (15)	0.0438 (16)	0.0041 (13)	0.0172 (13)	0.0011 (13)
C8	0.0400 (17)	0.0393 (16)	0.0336 (14)	0.0029 (13)	0.0128 (12)	0.0023 (12)
C9	0.0426 (19)	0.0434 (18)	0.0506 (18)	0.0012 (15)	0.0090 (14)	0.0056 (14)
C10	0.044 (2)	0.068 (2)	0.064 (2)	0.0131 (18)	0.0096 (16)	0.0015 (19)
C11	0.042 (2)	0.073 (3)	0.071 (2)	−0.007 (2)	0.0159 (17)	0.003 (2)
C12	0.054 (2)	0.055 (2)	0.065 (2)	−0.0116 (18)	0.0231 (17)	0.0018 (18)
C13	0.0457 (19)	0.0384 (16)	0.0527 (19)	−0.0038 (15)	0.0185 (15)	0.0002 (14)
Cl4	0.0492 (5)	0.0352 (4)	0.1229 (9)	0.0110 (4)	0.0283 (5)	0.0141 (5)
Cl5	0.0330 (4)	0.0743 (7)	0.1059 (8)	0.0019 (5)	0.0126 (4)	0.0079 (6)
Cl6	0.0455 (5)	0.0593 (6)	0.1157 (9)	0.0121 (5)	0.0322 (5)	−0.0116 (6)
O2	0.0459 (13)	0.0357 (11)	0.0453 (11)	0.0033 (10)	0.0119 (9)	0.0058 (9)
N2	0.0359 (14)	0.0321 (12)	0.0449 (14)	0.0007 (11)	0.0065 (11)	0.0086 (10)
C14	0.0328 (15)	0.0351 (15)	0.0367 (14)	−0.0006 (12)	0.0119 (11)	0.0055 (12)
C15	0.0423 (18)	0.0341 (16)	0.0485 (17)	0.0067 (14)	0.0184 (14)	0.0075 (13)
C16	0.0329 (16)	0.0432 (17)	0.0510 (18)	−0.0021 (13)	0.0161 (14)	0.0028 (14)
C17	0.047 (2)	0.0457 (18)	0.0525 (19)	−0.0114 (16)	0.0185 (15)	−0.0047 (15)
C18	0.054 (2)	0.0301 (15)	0.0572 (19)	0.0024 (14)	0.0201 (16)	−0.0038 (14)
C19	0.0352 (16)	0.0394 (16)	0.0454 (17)	0.0053 (14)	0.0101 (13)	0.0010 (13)
C20	0.0359 (16)	0.0339 (14)	0.0383 (15)	0.0072 (13)	0.0184 (12)	0.0006 (12)
C21	0.0363 (16)	0.0335 (15)	0.0374 (15)	0.0019 (13)	0.0111 (12)	−0.0031 (12)
C22	0.0395 (18)	0.0407 (17)	0.0472 (17)	0.0038 (14)	0.0133 (13)	−0.0028 (14)
C23	0.043 (2)	0.067 (2)	0.060 (2)	−0.0104 (19)	0.0078 (16)	−0.0007 (19)
C24	0.077 (3)	0.0399 (19)	0.054 (2)	−0.0137 (19)	−0.0003 (19)	0.0006 (15)
C25	0.074 (3)	0.0357 (17)	0.0509 (19)	0.0061 (18)	−0.0013 (17)	−0.0010 (14)
C26	0.0487 (19)	0.0397 (16)	0.0413 (17)	0.0060 (15)	0.0052 (14)	0.0001 (14)

### Geometric parameters (Å, °)

**Table:**

Cl1—C2	1.728 (3)	Cl4—C15	1.735 (3)
Cl2—C3	1.730 (4)	Cl5—C16	1.735 (3)
Cl3—C9	1.739 (4)	Cl6—C22	1.733 (3)
O1—C7	1.226 (4)	O2—C20	1.222 (4)
N1—C7	1.353 (4)	N2—C20	1.371 (4)
N1—C1	1.411 (4)	N2—C14	1.409 (4)
N1—H1N	0.8600	N2—H2N	0.8600
C1—C6	1.391 (5)	C14—C19	1.395 (4)
C1—C2	1.401 (4)	C14—C15	1.396 (4)
C2—C3	1.397 (5)	C15—C16	1.377 (5)
C3—C4	1.373 (5)	C16—C17	1.397 (5)
C4—C5	1.384 (6)	C17—C18	1.380 (5)
C4—H4	0.9300	C17—H17	0.9300
C5—C6	1.384 (6)	C18—C19	1.381 (5)
C5—H5	0.9300	C18—H18	0.9300
C6—H6	0.9300	C19—H19	0.9300
C7—C8	1.507 (5)	C20—C21	1.493 (4)
C8—C13	1.390 (5)	C21—C26	1.393 (4)
C8—C9	1.402 (5)	C21—C22	1.402 (4)
C9—C10	1.392 (6)	C22—C23	1.382 (5)
C10—C11	1.371 (6)	C23—C24	1.382 (6)
C10—H10	0.9300	C23—H23	0.9300
C11—C12	1.370 (6)	C24—C25	1.376 (7)
C11—H11	0.9300	C24—H24	0.9300
C12—C13	1.392 (5)	C25—C26	1.375 (5)
C12—H12	0.9300	C25—H25	0.9300
C13—H13	0.9300	C26—H26	0.9300
			
C7—N1—C1	126.2 (3)	C20—N2—C14	124.1 (3)
C7—N1—H1N	116.9	C20—N2—H2N	118.0
C1—N1—H1N	116.9	C14—N2—H2N	118.0
C6—C1—C2	118.7 (3)	C19—C14—C15	118.0 (3)
C6—C1—N1	122.2 (3)	C19—C14—N2	121.5 (3)
C2—C1—N1	119.1 (3)	C15—C14—N2	120.5 (3)
C3—C2—C1	120.2 (3)	C16—C15—C14	120.8 (3)
C3—C2—Cl1	119.9 (3)	C16—C15—Cl4	120.1 (3)
C1—C2—Cl1	119.9 (3)	C14—C15—Cl4	119.0 (3)
C4—C3—C2	120.3 (3)	C15—C16—C17	120.8 (3)
C4—C3—Cl2	119.1 (3)	C15—C16—Cl5	121.3 (3)
C2—C3—Cl2	120.6 (3)	C17—C16—Cl5	117.9 (3)
C3—C4—C5	119.9 (3)	C18—C17—C16	118.4 (3)
C3—C4—H4	120.0	C18—C17—H17	120.8
C5—C4—H4	120.0	C16—C17—H17	120.8
C6—C5—C4	120.4 (4)	C17—C18—C19	121.1 (3)
C6—C5—H5	119.8	C17—C18—H18	119.4
C4—C5—H5	119.8	C19—C18—H18	119.4
C5—C6—C1	120.6 (4)	C18—C19—C14	120.8 (3)
C5—C6—H6	119.7	C18—C19—H19	119.6
C1—C6—H6	119.7	C14—C19—H19	119.6
O1—C7—N1	122.3 (3)	O2—C20—N2	123.1 (3)
O1—C7—C8	120.4 (3)	O2—C20—C21	122.6 (3)
N1—C7—C8	117.1 (3)	N2—C20—C21	114.2 (2)
C13—C8—C9	117.5 (3)	C26—C21—C22	117.9 (3)
C13—C8—C7	116.1 (3)	C26—C21—C20	120.0 (3)
C9—C8—C7	126.4 (3)	C22—C21—C20	122.0 (3)
C10—C9—C8	121.0 (3)	C23—C22—C21	120.9 (3)
C10—C9—Cl3	117.6 (3)	C23—C22—Cl6	118.0 (3)
C8—C9—Cl3	121.3 (3)	C21—C22—Cl6	121.1 (3)
C11—C10—C9	119.8 (4)	C22—C23—C24	119.7 (4)
C11—C10—H10	120.1	C22—C23—H23	120.2
C9—C10—H10	120.1	C24—C23—H23	120.2
C12—C11—C10	120.7 (4)	C25—C24—C23	120.3 (4)
C12—C11—H11	119.6	C25—C24—H24	119.9
C10—C11—H11	119.6	C23—C24—H24	119.9
C11—C12—C13	119.7 (4)	C26—C25—C24	120.1 (4)
C11—C12—H12	120.1	C26—C25—H25	119.9
C13—C12—H12	120.1	C24—C25—H25	119.9
C8—C13—C12	121.3 (3)	C25—C26—C21	121.1 (3)
C8—C13—H13	119.4	C25—C26—H26	119.5
C12—C13—H13	119.4	C21—C26—H26	119.5
			
C7—N1—C1—C6	36.2 (5)	C20—N2—C14—C19	−43.2 (4)
C7—N1—C1—C2	−145.3 (3)	C20—N2—C14—C15	136.7 (3)
C6—C1—C2—C3	−0.5 (5)	C19—C14—C15—C16	−0.8 (4)
N1—C1—C2—C3	−179.1 (3)	N2—C14—C15—C16	179.2 (3)
C6—C1—C2—Cl1	179.5 (3)	C19—C14—C15—Cl4	179.2 (2)
N1—C1—C2—Cl1	0.8 (4)	N2—C14—C15—Cl4	−0.7 (4)
C1—C2—C3—C4	−0.6 (5)	C14—C15—C16—C17	2.3 (5)
Cl1—C2—C3—C4	179.5 (3)	Cl4—C15—C16—C17	−177.8 (3)
C1—C2—C3—Cl2	−179.4 (3)	C14—C15—C16—Cl5	−178.9 (2)
Cl1—C2—C3—Cl2	0.7 (4)	Cl4—C15—C16—Cl5	1.1 (4)
C2—C3—C4—C5	1.0 (6)	C15—C16—C17—C18	−2.6 (5)
Cl2—C3—C4—C5	179.9 (3)	Cl5—C16—C17—C18	178.5 (3)
C3—C4—C5—C6	−0.5 (6)	C16—C17—C18—C19	1.6 (5)
C4—C5—C6—C1	−0.6 (6)	C17—C18—C19—C14	−0.3 (5)
C2—C1—C6—C5	1.0 (5)	C15—C14—C19—C18	−0.1 (5)
N1—C1—C6—C5	179.6 (3)	N2—C14—C19—C18	179.8 (3)
C1—N1—C7—O1	0.1 (5)	C14—N2—C20—O2	3.4 (5)
C1—N1—C7—C8	−175.7 (3)	C14—N2—C20—C21	−173.0 (3)
O1—C7—C8—C13	−39.9 (4)	O2—C20—C21—C26	−128.9 (3)
N1—C7—C8—C13	135.9 (3)	N2—C20—C21—C26	47.5 (4)
O1—C7—C8—C9	140.8 (3)	O2—C20—C21—C22	48.7 (4)
N1—C7—C8—C9	−43.4 (4)	N2—C20—C21—C22	−134.9 (3)
C13—C8—C9—C10	−1.1 (5)	C26—C21—C22—C23	−1.8 (5)
C7—C8—C9—C10	178.2 (3)	C20—C21—C22—C23	−179.4 (3)
C13—C8—C9—Cl3	175.0 (2)	C26—C21—C22—Cl6	178.6 (2)
C7—C8—C9—Cl3	−5.7 (5)	C20—C21—C22—Cl6	1.0 (4)
C8—C9—C10—C11	0.3 (6)	C21—C22—C23—C24	1.1 (5)
Cl3—C9—C10—C11	−175.8 (3)	Cl6—C22—C23—C24	−179.3 (3)
C9—C10—C11—C12	0.4 (6)	C22—C23—C24—C25	0.6 (6)
C10—C11—C12—C13	−0.3 (6)	C23—C24—C25—C26	−1.7 (6)
C9—C8—C13—C12	1.2 (5)	C24—C25—C26—C21	1.0 (6)
C7—C8—C13—C12	−178.2 (3)	C22—C21—C26—C25	0.7 (5)
C11—C12—C13—C8	−0.5 (6)	C20—C21—C26—C25	178.4 (3)

### Hydrogen-bond geometry (Å, °)

**Table:**

*D*—H···*A*	*D*—H	H···*A*	*D*···*A*	*D*—H···*A*
N1—H1N···O2	0.86	2.16	2.850 (3)	137
N1—H1N···Cl3	0.86	2.64	3.114 (3)	116
N2—H2N···O1^i^	0.86	2.05	2.896 (3)	167

Symmetry codes: (i) *x*, −*y*+1, *z*−1/2.
